# Methicillin-Resistant and Methicillin-Susceptible *Staphylococcus* from Vervet Monkeys (*Chlorocebus sabaeus*) in Saint Kitts

**DOI:** 10.3390/antibiotics10030290

**Published:** 2021-03-10

**Authors:** Andreas Hoefer, Filip Boyen, Amy Beierschmitt, Arshnee Moodley, Marilyn C. Roberts, Patrick Butaye

**Affiliations:** 1 School of Veterinary Medicine, Ross University, West Farm 000265, Saint Kitts and Nevis; andreashoefer@hotmail.com (A.H.); ABeierschmitt@rossvet.edu.kn (A.B.); 2 Department of Pathology, Bacteriology and Avian Diseases, Faculty of Veterinary Medicine, Ghent University, 9000 Merelbeke, Belgium; filip.boyen@Ugent.be; 3 Behavioral Science Foundation, Basseterre KN 0101, Saint Kitts and Nevis; 4 Department of Veterinary and Animal Sciences, Faculty of Health and Medical Sciences, University of Copenhagen, 1165 Copenhagen, Denmark; asm@sund.ku.dk or; 5 CGIAR AMR Hub, International Livestock Research Institute, Nairobi, Kenya; 6 Department of Environmental and Occupational Health, School of Public Health, University of Washington, Seattle, WA 98195, USA; marilynr@uw.edu

**Keywords:** methicillin resistance, *Staphylococcus*, whole-genome sequencing, monkey, vervet, *Chlorocebus sabaeus*

## Abstract

Antimicrobial resistance has been described in all ecosystems, including wildlife. Here we investigated the presence of methicillin-resistant and susceptible staphylococci in both colony-born and wild vervet monkeys (*Chlorocebus sabaeus*). Through selective isolation, PCR, MALDI-TOF, and whole-genome sequencing, methicillin-resistant and susceptible *Staphylococcus* spp. isolated from vervet monkeys were characterized. We obtained putatively methicillin-resistant staphylococci from 29 of the 34 nasal samples collected. Strains were identified by MALDI-TOF analysis. *Staphylococcus cohnii* (*n* = 15) was the most commonly isolated species, while nine other species were isolated one or two times. PCR analysis indicated that eight [28%] strains were *mecA* positive. The whole-genome sequencing [WGS] included eight methicillin-resistant strains (*S. epidermidis* (*n* = 2), *S. cohnii* (*n* = 3), *S. arlettae* (*n* = 2) and *S. hominis* (*n* = 1)), nine additional *S. cohnii* strains and two strains that could not be identified by MALDI-TOF, but genetically characterized as one *S. cohnii* and one *S. warneri*. Different resistance genes carried by different mobile genetic elements, mainly *blaZ* (*n* = 10) and *tet*(K) (*n* = 5) were found, while *msr*(A), *cat*, *fosB*, *dfrG*, *erm*(C), *mph*(C) and *str* were identified in one to three strains. Phylogenetic analysis of the *S. cohnii* strains based on SNPs indicated four clusters associated with colony born or wild. In addition, one singleton *S. cohnii* isolated did not form a separate group and clustered within other *S. cohnii* strains submitted to the NCBI. In this study, we demonstrated the presence of AMR and mobile genetic elements to both colony-born and wild vervet monkeys. We also identified a previously undescribed prevalence of *S. cohnii* in the nasal flora of these monkeys, which merits further investigation.

## 1. Introduction

Methicillin-resistant *Staphylococcus aureus* (MRSA) is a major health problem in humans, while in animals, it remains limited to sporadic cases [1]. That being said, the potential for zoonotic transmission of MRSA has been well documented [2,3]. In animals, methicillin-resistant *S. pseudintermedius* is becoming an imminent health problem in dogs [4]. Specifically, in animals, methicillin-resistance has not been limited to coagulase-positive staphylococci [5,6]. Transfer of the SCC*mec* element can be mediated by bacteriophages [7], which, based on the zoonotic potential, makes the presence of methicillin-resistance in animal staphylococci a matter of public health concern [2].

Currently, 71 recognized staphylococcal species and 30 subspecies have been described (http://www.dsmz.de/bacterial-diversity/prokaryotic-nomenclature-up-to-date, last accessed on 8 March 2021). However, little is known about the presence of staphylococci in monkeys. Moreover, most studies on staphylococci in monkeys were focused on the isolation of MRSA and *S. aureus*. We could not find any study dealing with vervet monkeys and staphylococci. In other monkey species, few studies were done on the presence of staphylococci, like a study in gorillas where several staphylococcal species were found in the feces [8] as well as on the conjunctiva [9]. Staphylococci have also been found in the vagina of squirrel monkeys [10], where a new species was discovered [11] and in the nasal, oral, rectal and vaginal flora of tamarins [12,13]. However, the information is fragmentary and does not always include the susceptibility of the strains. Only two studies also reported on antimicrobial resistance, and 13% of the staphylococci from squirrel monkeys proved to be methicillin-resistant [10,13].

In monkeys, as well as other wildlife species, MRSA has been frequently described [2,3,9,14,15,16,17]. However, little is known about methicillin-resistance harbored by other staphylococcal species in monkeys and the zoonotic potential of these methicillin-resistant staphylococci. Therefore, we performed a study on Saint Kitts Island to further investigate the presence of methicillin-resistance in monkey staphylococci and to evaluate their potential to act as a natural reservoir for clinically significant resistance determinants. Saint Kitts is a small island nation in which the monkey population is estimated to exceed the human population leading to frequent direct and indirect contact between humans and monkeys. While the prevalence of MRSA in humans in the hospital is well described, little is known about this pathogen in the wild and captive monkey populations of the island [18]. Such a large population of monkeys on a small island makes this study very well suited for One Health-oriented investigations.

Since methicillin in humans appears to be highly prevalent in humans in Saint Kitts, we hypothesize that this resistance is more widespread in the environment.

In this research, the aim was to determine the presence and characteristics of *Staphylococcus* spp., isolated from nasal swab samples of vervet monkeys and using cefoxitin supplemented selective media. A second objective was to determine whether there were differences in the frequency of species and diversity of strains in the captive and free-living vervet monkey populations on the island of Saint Kitts

## 2. Results

Thirty-four samples were taken, of which 21 originated from wild-caught animals and 13 from animals living in captivity. Of the 34 samples, using all methods together, five were negative for staphylococci. In the 29 remaining cultures, the multiplex PCR indicated the isolated strains were staphylococci, and two were *S. aureus*. Only eight strains showed the presence of the *mecA* gene (Table 1), five from captive animals (38.5% CI: 13.9–68%) and three from wild animals (14.3% CI: 3–36%). This difference was not statistically significant. MALDI-TOF could not identify two strains, which were subsequently identified by WGS and Kmer analysis. Finally, we detected *S. cohnii* (*n* = 15), *S. epidermidis* (*n* = 2), *S. pettenkoferi* (*n* = 1), *S. kloosii* (*n* = 1), *S. saprophyticus* (*n* = 1), *S. arlettae* (*n* = 2), *S. aureus* (*n* = 2), *S. xylosus* (*n* = 2) and *S. hominis* (*n* = 2) and *S. warneri* (*n* = 1) (Table 1).

A selection of 19 strains (Table 1) was retained for WGS analysis based on the presence of *mecA*, no identification by MALDI-TOF and the most prevalent species. Sequence quality control parameters can be found in Table 2. All trimmed raw reads were deposited in NCBI BioProject database and assigned BioProject Temporary SubmissionID PRJNA647767, with accession numbers https://www.ncbi.nlm.nih.gov/biosample/15636413, https://www.ncbi.nlm.nih.gov/biosample/15636414 https://www.ncbi.nlm.nih.gov/biosample/15636415, https://www.ncbi.nlm.nih.gov/biosample/15636416, https://www.ncbi.nlm.nih.gov/biosample/15636417, https://www.ncbi.nlm.nih.gov/biosample/15636418, https://www.ncbi.nlm.nih.gov/biosample/15636419, https://www.ncbi.nlm.nih.gov/biosample/15636420, https://www.ncbi.nlm.nih.gov/biosample/15636421, https://www.ncbi.nlm.nih.gov/biosample/15636422, https://www.ncbi.nlm.nih.gov/biosample/15636423, https://www.ncbi.nlm.nih.gov/biosample/15636424, https://www.ncbi.nlm.nih.gov/biosample/15636425, https://www.ncbi.nlm.nih.gov/biosample/15636426, https://www.ncbi.nlm.nih.gov/biosample/15636427, https://www.ncbi.nlm.nih.gov/biosample/15636428, https://www.ncbi.nlm.nih.gov/biosample/15636429, https://www.ncbi.nlm.nih.gov/biosample/15636430, https://www.ncbi.nlm.nih.gov/biosample/15636431.

By PCR, the *mecA* gene was identified in eight strains; however, in four cases, ResFinder did not detect the gene, while RAST analysis did not find the gene in two strains. In these two strains, only sequences potentially associated with SCC*mec* elements could be found. In the two strains where we were able to find the *mecA* gene through RAST, the gene was located on a very small contig.

We could identify two different SCC*mec* elements in the collection of strains. The two *S. epidermidis* strains were SCC*mec* type V(5C2), and one *S. cohnii* strain contained type SCC*mec* type III (3A). Frequently, the SCC*mec* could not be determined, probably due to fragmentation and gaps in the sequences. On the other hand, in some *mecA* negative strains, fragments of SCC*mec* were identified by SCC*mec*Finder, namely ccrA1:4, part of the LGA251SCC*mec* in 7 *S. cohnii* strains, and in six instances also with a part of the subtype-Vc(5C2&5) (Table 1). Further analysis of the contig containing the *ccrA1;4* genes indicated that the full contig of 9034 base pairs was also present in an *S. nepalensis* strain with a coverage of 71% and 95.78% identity. The *ccrA* gene detected is the *ccrA* gene present in SCC*mec* IX. The part containing a portion of the subtype-Vc(5C2&5) was small and related to the presence of the *czr* gene, encoding zinc resistance, typically associated with LA-MRSA ST398 [1].

In both *S. arlettae* isolates, *mecA* was detected by PCR, while ResFinder could only detect the gene in one of the strains. RAST analysis, however, demonstrated the presence of the *mecA* gene in both strains. No SCC*mec* could be identified as the contigs on which the *mecA* gene was located were short; in one strain, it was only the *mecA* gene on the contig and in the other strain, there were only two additional genes, previously associated with SCC*mec,* but of insufficient length to determine the type.

In the *S. hominis* strain, the gene was found with a *mecI* and *mecR* gene in one node, while the *ccrC1* allele was found on another contig and is associated with SCC*mec* type II. No definitive SCC*mec* could be determined.

The resistance genes found by ResFinder were *blaZ* in ten strains, *tet*(K) in five, the *msr(A)* gene in one and the *mph*(C) gene in three, the *cat* gene in one, the *fosB* genes were associated with the two *S. epidermidis* strains and those two strains carried the *dfrG* gene. The *erm*(C) gene was found in one of the two *S. epidermidis* strains. Finally, the *str* gene was found in one of the *S. arlettae* strains (Table 1).

Tetracycline resistance genes *tet*(K) were located on plasmids, but it was not clear which ones. RAST analysis showed that the *tet*(K) gene was located on the same plasmid in cluster three *S. cohnii* strains but was different in cluster two *S. cohnii* strains. The BLAST analysis showed that the replication initiation protein was most similar to the *S. aureus* subsp. *aureus* strain ST20071176 plasmid pT45. The next gene, a type I restriction–modification system, specificity subunit S, had very little homology with other chromosomal genes. The plasmid recombination, *mobE* mobilization protein gene was very similar to the *mobE* gene of *S. warneri* strain WB224 plasmid pWB224_2, and the downstream *mobE* gene was also 100% identical in that plasmid and several other plasmids of staphylococci. These genes were followed by the *tet*(K) gene, and the next gene was another replication initiation protein identical to *S. aureus* strain SR153 plasmid pSR02 replication initiation protein. This was then followed by another type I restriction–modification system (with low similarity) and another *mobE* gene, similar to the first one. The other location of the *tet*(K) gene was surrounded by two replication initiation proteins and two hypothetical proteins. *tet*(K) is frequently associated with IS*257*; however, this was not the case in our study, as we could not find any insertion sequence in the *tet*(K) positive strains. None of these sequences were identified as plasmids by the PlasmidFinder. Similarly, chloramphenicol resistance was located on a plasmid as determined by the ResFinder and RAST analysis. The *mph*(C) gene could not be associated with a mobile genetic element, and the *blaZ* gene was associated with a Tn*552*-like element in all but the *S. hominis* strain. (Table 1).

The *erm*(C) gene was associated with a gene encoding a replication and maintenance protein. BLAST analysis of this gene showed that it was associated with many staphylococcal plasmids; the contig was, however, too small to find a more exact location.

Several plasmids were identified in the strains using PlasmidFinder (Table 1). Four strains did not contain any plasmids, three *S. cohnii* strains, of which two had no resistance genes, and one strain carried the *blaZ* and *tet*(K), which can be found on the chromosome. The other strain was the *mecA* positive *S. arlettae* carrying no other resistance genes. In five strains, two plasmids were detected in each. Eight different replicon types were identified (Table 1). The replicon type 7a was most common (*n* = 8), though they were not all associated with the same plasmid types. The other seven replicon types were only found one to four times only.

**Figure 1:**
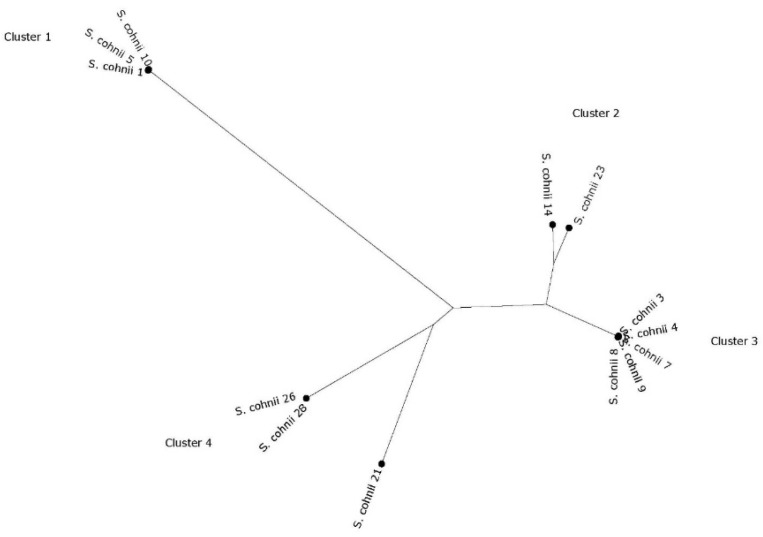
Core genome phylogeny of the vervet *S. cohnii* isolates. Phylogeny was based on SNP differences.

**Figure 2:**
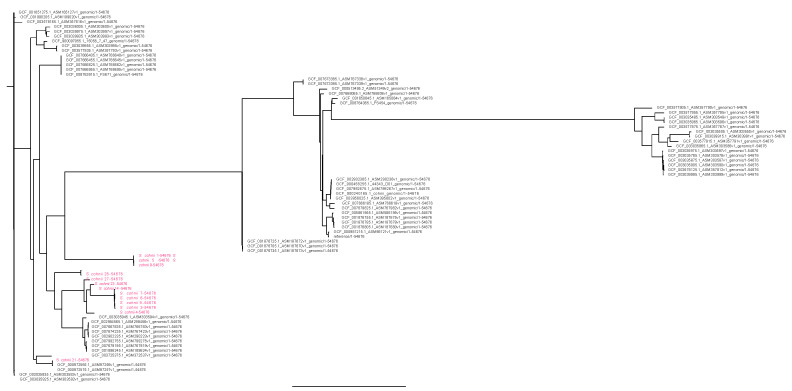
Rooted phylogenies of all sequenced *S. cohnii* isolates present in NCBI on 29 April 2020.

The two *S. epidermidis* strains were both ST210, and the single *S. hominis* strain had an unknown MLST profile but with a close match to ST13. No MLST scheme was available for the other species. Further phylogenetic analysis using CSIPhylogeny demonstrated that the two *S. epidermidis* strains were very similar but not identical with 81 SNP differences (data not shown). Phylogenetic analysis of the *S. cohnii* strain identified four clusters and a singleton (Figure 1). In cluster one, with three nearly identical strains (between 50 and 68 SNPs different), one strain contained SCC*mec* type III (3A), while in two others, the *mecA* gene was found, but the SCC*mec* type could not be determined. Cluster two contained non-identical strains with a difference of 1368 SNPs. Cluster three is the largest cluster and contained five strains. Differences in SNPs were between 41 and 104. All harbored the same fragments of SCC*mec* as determined by SCC*mec*Finder and RAST analysis. However, no *mecA* gene could be detected in any of the analyses. Cluster four contained two very similar strains with 44 SNP differences, and there was one singleton (strain 21) containing different parts of SCC*mec*, but it remains undefined. Typically, each cluster was composed of strains only belonging to wild or colony monkeys, indicating a different evolution of the strains with little mixing between wild and colony monkeys. Looking at the phylogenetic tree constructed with all at NCBI available sequenced *S. cohnii* strains [sequences of 63 strains were available and downloaded on April 29, 2020), the strain clustered among all different *S. cohnii* strains without any clear pattern (Figure 2). No virulence genes were detected in the *S. epidermidis* strains.

## 3. Discussion

To our knowledge, this is the first study that investigates methicillin-resistant and methicillin-susceptible staphylococcal species isolated from the nasal passage of monkeys. We sampled both wild and captive animals, though we could not detect a significant difference in the prevalence between the two populations. The wild animals were captured at various sites throughout the Island, while the colony-born vervets were living in closer contact. The wild animals had never received antimicrobial treatments, yet their staphylococci harbored a variety of resistance genes, probably picked up from other sources, including human interaction and the environment. However, the presence of the *mec*A genes in wild animals suggests that the *mecA* gene is widespread in Saint Kitts and Nevis environments. This may have been through human contact as the prevalence of MRSA in the hospital is very high, with 45% of the *S. aureus* infections being caused by MRSA or by feeding in human garbage and handouts like in Nepal [2,16]. There are, however, no data on the presence of methicillin-resistant non-*aureus* staphylococci from humans in Saint Kitts since data on these types of isolates are not generally collected. These are the first data on staphylococci from animals in Saint Kitts. This may suggest that methicillin-resistant non-*aureus* staphylococci could act as a reservoir for methicillin-resistance determinants.

One of the first surprises was that several of the strains were not methicillin-resistant despite isolation on selective media. This may be due to the presence of the *blaZ* gene that may have influenced the recovery of such strains; however, that is certainly not a valid hypothesis for all of the strains. It remains unclear what is the reason for the growth of these strains on the selective media and warrants further study. The *mecA* gene was also identified more frequently by PCR than by WGS, though depending on the analysis method, we could find more *mecA* genes using RAST analysis rather than ResFinder. This is most probably because RAST has no selection parameters, while in ResFinder, we used the standard parameters. Nevertheless, when the PCR is positive, regardless of the WGS analysis, the strain was regarded as *mecA* positive as the WGS sequences are generally incomplete.

In this study, a little more than half of the staphylococci isolated were *S. cohnii*. There is, in general, very limited knowledge about *S. cohnii*. *S. cohnii* has been isolated from different animal species without pathology (as dogs, cattle, pet animals), from environmental samples and on rare occasions, *S. cohnii* has been isolated from infections in humans, indicating that some strains may have some pathogenic potential [19,20,21,22]. It has been shown before that this species can be intrinsically resistant to penicillins and negative for all known β-lactam resistance genes [23]. Furthermore, taking into account the selective isolation method used for isolating these strains, it may have created a bias for the selection of antibiotic-resistant isolates. Nevertheless, there are indications that there is homogeneity in the strains as they are frequently clustered as closely related (Figure 1). It was striking to see that the strains from wild animals had separate clusters from the colony animals, indicating little mixing of the animals and the strains having a different evolution. Nevertheless, looking at all sequenced *S. cohnii* strains available in the NCBI database, they do not form a specific Saint Kitts vervet cluster in the currently available sequenced strain collection. It would be interesting to see what the prevalence of *S. cohnii* in other populations is, including humans, in Saint Kitts.

In the pubMLST database (https://pubmlst.org/bigsdb?db=pubmlst_sepidermidis_isolates&page=query, last accessed June 2020), four other *S. epidermidis* strain have been sequence typed as ST210 strains, strains originating from India, South Korea, and Portugal. All were of human origin, though it should be noted that few animal strains are included in this database, and most of the animal strains are from cases of mastitis in cattle. Former studies have shown that there is a high diversity of strains and no clear delineation between human and animal strains, though this study did not include monkey strains [24]. Moreover, to the best of our knowledge, this is the first study characterizing methicillin-resistant *S. epidermidis* from monkeys. We could not find any virulence genes associated with either human or animal strains.

Few SCC*mec* types could be detected. Moreover, we found different fragments pertaining to SCC*mec* in several of the strains, lacking the *mecA* gene. Looking for SCC*mec,* associated genes on the RAST annotated contigs did not solve the problem as exemplified in the *S. hominis* strain. This could also indicate that there remain uncharacterized SCC*mec* elements in the strains. The significance of these findings is not clear. Further in-depth sequencing and gap closing could give more insights into this finding. A recent study showed the high prevalence (45%) of the MRSA USA 300 strain among *S. aureus* infections in Saint Kitts. However, this strain carries SCC*mec* IVa, which was not found in this study [18].

The *tet*(K) gene was clearly associated with a plasmid; we could, however not determine, which plasmid it was. Homology searches did not reveal any specific plasmid. *tet*(K) in staphylococci is frequently associated with an insertion element, though we could not find any linked insertion sequence either.

The *msr*(A) gene codes for an ABC-F protein, which confers macrolide and streptogramin B resistance. Recently, the action has been shown to be due to ribosomal protection by these proteins [25,26]. The *msr*(A) gene mediates resistance to macrolides and streptogramin B antibiotics, but not to 16-membered macrolides and lincosamides. This gene has been associated with plasmids, frequently together with *erm* genes. However, in this *S. hominis* strain, we could not find any plasmid, nor an *erm* gene. The chloramphenicol resistance gene was associated with a plasmid that was not identified by the PlasmidFinder software. PC221 is a Rolling-circle plasmid that has been found in several staphylococci [27]. Other elements involved in the mobility of resistance was the Tn*552* transposon associated with *blaZ*.

## 4. Materials and Methods

This study was approved by the IACUC of RUSVM with file number 17.04.20.

### 4.1. Sampling

Thirty-four vervet monkeys (*Chlorocebus sabaeus*) were sampled by nose swabs (BBL CultureSwab plus Amies Medium, Becton Dickinson, New Jersey, US). Of these 34, 13 were animals living in captivity for many generations in the Behavioral Science Foundation, and 21 wild monkeys were caught on the island of Saint Kitts. The captive animals were between 3 and 15 years of age and of both sexes. All were colony-born. Animals did not receive antibiotics prior to this study and were swabbed during routine husbandry and thus randomly selected. The animals are housed in outdoor cages, grouped to the same sex or in breeding groups. Wild animals were trapped in funnel-type traps by professional trappers and according to the animal welfare regulations (and approved by the BSF IACUC). After trapping, they were brought to the facility for quarantine. Estimated ages were between 3 and 15 years. They were trapped at various parts of the island of Saint Kitts, from rural/farming areas. In general, they stay away from humans but share territory at different times; they do not get into contact with sewage nor garbage as this is not their typical behavior. Nasal swabs were taken and transported to the lab for further analysis within 4 h.

### 4.2. Isolation of Methicillin-Resistant Staphylococci

**Figure 3:**
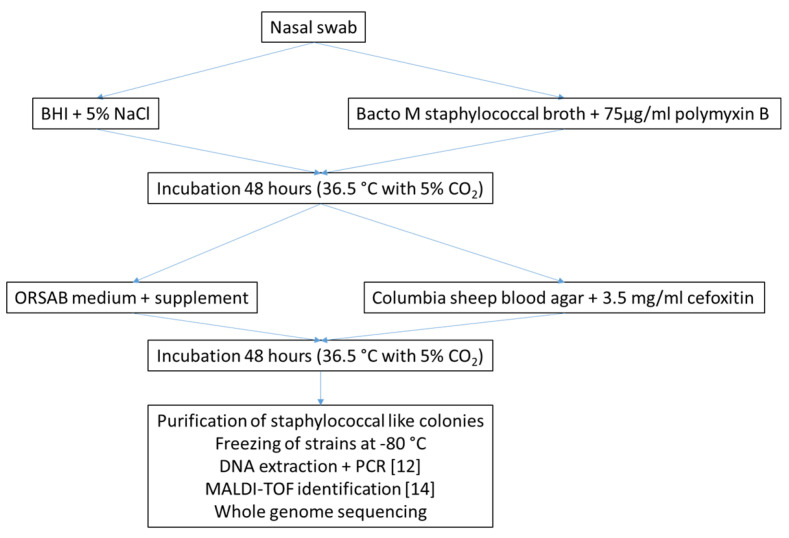
Isolation and identification strategy.

Isolation of methicillin-resistant staphylococci was performed as described before [28], with some adaptations. Briefly, the nasal swabs were inoculated in two different enrichment media, BHI supplemented with 5% NaCl and Bacto M staphylococcal broth (Becton Dickinson, NJ, USA) supplemented with 75 µg/mL of polymyxin B (Sigma-Aldrich, Saint Louis, MO, USA) and incubated for 48 h at 36.5 °C and 5% CO_2_. Upon growth in these media, a loopful was inoculated on selective plates. One ORSAB plate with SR0195 selective supplement (Oxoid, Basingstoke, UK) for the isolation of MRSA was inoculated, as well as one Columbia sheep blood agar plate supplemented with 3.5 µg/mL of cefoxitin (Sigma, Basingstoke, UK). Colonies resembling staphylococcal morphology were purified on blood agar plates (Figure 3).

### 4.3. Bacterial Identification

Purified strains were initially identified with a PCR for the staphylococcal 16S rRNA gene, the *nuc* gene, specific for *S. aureus,* as well as the *mecA* gene [29]. DNA was extracted as previously described [28]. Further identification of the strains was performed using MALDI-TOF Biotyper (Bruker Daltonics, Germany), as previously described [30]. Final identification was based on the whole-genome sequence analysis.

### 4.4. Whole-Genome Sequencing

Overnight cultures were grown in tryptic soy broth at 37 °C with 200-rpm shaking. Genomic DNA from the staphylococcal strains was isolated using the DNeasy Blood and Tissue kit (Qiagen). DNA purity and concentration were determined using the Nanodrop and Qubit instruments, respectively. Sequencing library preparation was done using the Nextera XT kit and sequenced on a MiSeq using a paired-end 2 × 250 bp sequencing strategy, all following standard Illumina protocols (Illumina, Inc., San Diego, CA, USA).

Adapters were trimmed from the raw reads using the MiSeq program. Initial analyses (de novo assembly) and quality control were performed using Velvet.

The following analysis performed with pipelines from the Center for Genomic Epidemiology (http://www.genomicepidemiology.org/, last accessed 9 March 2021) were used: Kmer analysis to confirm the species identification (KmerFinder), ResFinder v.3.0 for the detection of resistance genes, PlasmidFinder v2.0 for the detection of plasmid replicons, SCC*mec* was identified using SCC*mec*Finder, MLST profiles were determined using “MLST”. Genomes of the *S. cohnii* strains isolated in this study were compared to the 64 *S. cohnii* strains submitted to the NCBI database using CSIPhylogeny for SNP analysis. Trees were constructed using Figtree V1.4.4 with standard settings.

Strains were annotated using the RAST server using standard settings. Specific contigs were inspected manually and in some cases compared with other sequences using BLAST analysis.

RAST analysis was performed for the detection of virulence genes in *S. epidermidis.* We specifically looked for the *ica*RADBC operon, the biofilm-related genes *embp*, *aap*, *bhp* and IS*256* [31].

### 4.5. Statistical Analysis

Groups were compared using Fisher’s exact test.

## 5. Conclusions

This is the first study into the methicillin-resistant staphylococcal flora of non-human primates. In this study, we isolated several methicillin-resistant staphylococcal species as well as susceptible staphylococci growing on selective methicillin-resistant plates. methicillin-resistance could not always be associated with an SCC*mec* element, while some strains lacking *mecA* contained parts of an SCC*mec* element. Whole-genome analysis showed that there were some well-known resistance genes present, but it could not always be determined with which exact mobile genetic element they were associated. The high number of *S. cohnii* in these animals requires further investigation on their importance in the nasal flora of vervet monkeys. The resistance genes in association with known mobile genetic elements indicate a potential spillover from other sources as the wild-caught animals do not receive antimicrobials. Our findings confirm a potential public health threat caused by these resistance genes found in pathogens demonstrated to have zoonotic potential.

## Figures and Tables

**Table 1:** Isolation results of the samples and DNA analysis of the strains.

Sample	Origin	*mecA*	Species	Resistance Genes ***	SCC*mec* Elements	Plasmids
1 **	Captive	+	*S. cohnii*	*blaZ*, *mph*(C)	SCC*mec*III(3A)	rep7a rep(pSBK203) U35036
2 **	Captive	+	*S. epidermidis*	*fosB*, *dfrG*, *erm*(C)	SCC*mec*V(5C2)	rep10 repL(pDLK1) GU562624, rep39 repA(SAP110A) GQ900465
3 **	Captive	-	*S. cohnii*	*blaZ*, *tet*(K)	ccrA1:4:LGA251:FR821779 subtype-Vc(5C2&5):10:AB505629	rep7a repC(pS0385p1) AM990993 rep21 rep(pSHaeA) AP006717
4 **	Captive	-	*S. cohnii*	*blaZ*, *tet*(K)	ccrA1:4:LGA251:FR821779 subtype-Vc(5C2&5):10:AB505629	rep7a repC(pS0385p1) AM990993/ repC(Cassette) AB037671
5 **	Captive	+	*S. cohnii*	*blaZ*, *mph*(C)		rep7a rep(pSBK203) U35036
6	Captive	-	*S. pettenkoferi*			none
7 **	Captive	-	*S. cohnii*	*blaZ*, *tet*(K)	ccrA1:4:LGA251:FR821779 subtype-Vc(5C2&5):10:AB505629	rep7a repC(pS0385p1) AM990993/repC(Cassette) AB037671/ORF(pKH1) SAU38656
8 **	Captive	-	*S. cohnii*	*blaZ*	ccrA1:4:LGA251:FR821779 subtype-Vc(5C2&5):10:AB505629	rep21 rep(pKH21) EU350088
9 **	Captive	-	*S. cohnii*	*blaZ*, *tet*(K)	ccrA1:4:LGA251:FR821779 subtype-Vc(5C2&5):10:AB505629	rep7a repC(pS0385p1) AM990993
10 **	Captive	+	*S. cohnii*	*blaZ*, *mph*(C)		none
11 **	Captive	-	S. warneri *	none		repUS35 A28412072(pvSw2) CP003671
12	Wild	-	*S. kloosii*			none
13	Wild	-	*S. saprophyticus*			none
14 **	Wild	-	*S. cohnii*	none	ccrB3:2:LGA251:FR821779	rep7a rep(pSBK203) U35036 rep21 rep(pKH21) EU350088
15	Wild	-	*S. xylosus*			none
16 **	Wild	+	*S. arlettae*	*str*		rep13 ORF(pC194) V01277
17	Wild	-	*S. aureus*			none
18 **	Wild	+	*S. arlettae*	none		none
19 **	Wild	+	*S. epidermidis*	*fosB*, *dfrG*	SCC*mec*V(5C2)	rep10 repL(pDLK1) GU562624 rep39 repA(SAP110A) GQ900465
20	Wild	-	*S. aureus*			
21 **	Wild	-	*S. cohnii*	none	ccrA1:4:LGA251:FR821779 subtype-Vc(5C2&5):10:AB505629	rep21 rep(pSHaeA) AP006717
22	Wild	-	*S. xylosus*			
23 **	Wild	-	*S. cohnii*	*blaZ*, *tet*(K), cat(pC211)		rep7a rep(pSBK203) U35036/repD(pTZ4) NC010111/ORF(pKH1) SAU38656
24	Wild	-	*S. cohnii*			
25 **	Wild	+	*S. hominis*	blaZ, *msr*(A)		rep7a repA(SAP105B) GQ900453
26 **	Captive	-	*S. cohnii*	none	ccrA1:3:JCSC6943:AB505628	none
27 **	Captive	-	S. cohnii *	none		none
28	Wild	NA	Negative			
29	Wild	NA	Negative			
30	Wild	NA	Negative			
31	Wild	-	*S. hominis*			
32	Wild	NA	Negative			
33	Wild	NA	Negative			
34	Wild	-	*S. cohnii*			

* only identified by WGS. ** WGS performed. *** resistance genes found with ResFinder.

**Table 2:** Quality parameters of the sequenced strains.

Sample	Number of Contigs	Mean Contig Length	Median Contig Length	Minimum Contig Length	Maximum Contig Length	Base Count	N50	Coverage
1	539	4548.83	2656	137	27,277	2,451,819	9591	29
2	469	196.37	2725	137	46,151	2,210,175	9380	31
3	514	4917.82	2570	137	35,832	2,527,758	10,396	29
4	676	152.46	2033	137	30,607	2,474,092	7349	27
5	524	187.09	2386	137	54,340	2,352,829	9277	27
7	554	4463.89	2638	137	37,050	2,472,996	8527	25
8	674	153.83	2184	137	38,142	2,488,125	7397	24
9	614	166.17	2257	137	43,634	2,448,661	8110	24
10	433	5385.63	3060	137	39,146	2,331,979	12,088	42
11	397	248.19	2854	137	44,913	2,364,664	12,992	35
14	543	4377.91	2462	137	37,869	2,377,204	8798	38
16	814	121.20	1738	137	24,999	2,367,457	5316	48
18	427	231.74	2963	137	55,529	2,374,768	11,863	39
19	375	6025.86	3340	137	38,343	2,259,697	12,172	40
21	435	238.79	2540	137	52,277	2,493,071	14,825	32
23	371	279.92	3240	137	71,562	2,492,410	14,287	32
25	386	220.87	2819	137	47,447	2,046,024	10,904	34
26	362	277.80	3243	137	56,956	2,413,478	15,510	29
27	441	228.60	3284	137	55,778	2,419,417	10,757	31

## Data Availability

NCBI BioProject database and assigned BioProject Temporary SubmissionID PRJNA647767, with accession numbers https://www.ncbi.nlm.nih.gov/biosample/15636413, https://www.ncbi.nlm.nih.gov/biosample/15636414, https://www.ncbi.nlm.nih.gov/biosample/15636415, https://www.ncbi.nlm.nih.gov/biosample/15636416, https://www.ncbi.nlm.nih.gov/biosample/15636417, https://www.ncbi.nlm.nih.gov/biosample/15636418, https://www.ncbi.nlm.nih.gov/biosample/15636419, https://www.ncbi.nlm.nih.gov/biosample/15636420, https://www.ncbi.nlm.nih.gov/biosample/15636421, https://www.ncbi.nlm.nih.gov/biosample/15636422, https://www.ncbi.nlm.nih.gov/biosample/15636423, https://www.ncbi.nlm.nih.gov/biosample/15636424, https://www.ncbi.nlm.nih.gov/biosample/15636425, https://www.ncbi.nlm.nih.gov/biosample/15636426, https://www.ncbi.nlm.nih.gov/biosample/15636427, https://www.ncbi.nlm.nih.gov/biosample/15636428, https://www.ncbi.nlm.nih.gov/biosample/15636429, https://www.ncbi.nlm.nih.gov/biosample/15636430, https://www.ncbi.nlm.nih.gov/biosample/15636431.
